# Target product profile: *Trypanosoma brucei gambiense* test to verify elimination

**DOI:** 10.2471/BLT.23.290177

**Published:** 2023-06-15

**Authors:** Gerardo Priotto, Jose R Franco, Veerle Lejon, Philippe Büscher, Enock Matovu, Joseph Ndung'u, Sylvain Biéler, Dieudonné Mumba, Nick Van Reet, Paul Verlé, Vincent Jamonneau, Pere P Simarro, Augustin Kadima Ebeja, Dieudonné Sankara, Daniel Argaw Dagne

**Affiliations:** a Control of Neglected Tropical Diseases, World Health Organization, Avenue Appia 20, 1211 Geneva 27, Switzerland.; b Institut de Recherche pour le Développement, CIRAD, University of Montpellier, France.; c Institute of Tropical Medicine, Antwerp, Belgium.; d College of Veterinary Medicine, Animal Resources and Biosecurity, Makerere University, Kampala, Uganda.; e Foundation for Innovative New Diagnostics–Kenya, Nairobi, Kenya.; f Neglected Tropical Diseases Programme, Foundation for Innovative New Diagnostics, Geneva, Switzerland.; g Department of Parasitology, Institut National de Recherche Biomédicale, Kinshasa, Democratic Republic of the Congo.; h Communicable Disease Unit, World Health Organization Regional Office for Africa, Brazzaville, Congo.

## Abstract

Human African trypanosomiasis is a life-threatening parasitic infection transmitted by the tsetse fly in sub-Saharan Africa. The most common form is caused by *Trypanosoma brucei gambiense*, with humans as the main reservoir. Diagnosis in the field requires microscopic examination performed by specifically trained personnel. After over two decades of sustained efforts, the incidence of the disease is strongly declining, and some historically endemic countries are no longer detecting cases. The World Health Organization (WHO) has targeted the elimination of transmission of gambiense human African trypanosomiasis by 2030, defined as zero autochthonous cases for at least five consecutive years. Endemic countries reaching this goal must maintain dedicated surveillance to detect re-emergence or re-introduction. With this new agenda, new tools are needed for verification of the absence of transmission. WHO has therefore developed a target product profile calling for development of a method for population-level cross-cutting surveillance of *T. b. gambiense* transmission. The method needs to be performed in national or sub-national reference laboratories, and to test in parallel numerous samples shipped from remote rural areas. Among other characteristics the product profile specifies: (i) a simple specimen collection procedure; (ii) no cold-chain requirement to transfer specimens to reference laboratories; (iii) high sensitivity and specificity; (iv) high-throughput, substantially automatized; (v) low cost per specimen, when analysed in large batches; and (vi) applicable also in animals.

## Résumé

La trypanosomiase humaine africaine est une infection parasitaire potentiellement mortelle transmise par la mouche tsé-tsé en Afrique subsaharienne. La forme la plus répandue est causée par *Trypanosoma brucei gambiense*, les humains constituant son principal réservoir. Établir un diagnostic sur le terrain nécessite un examen microscopique réalisé par du personnel formé à cet effet. Après plus de deux décennies d'efforts soutenus, l'incidence de la maladie diminue fortement et quelques pays historiquement endémiques ne découvrent plus aucun cas. L'objectif de l'Organisation mondiale de la Santé (OMS) est d'éliminer la transmission de la trypanosomiase humaine africaine à* T. b. gambiense* d'ici 2030, ce qui correspond à zéro cas autochtone pendant au moins cinq années consécutives. Les pays endémiques qui atteignent cet objectif doivent maintenir une surveillance spécifique afin de détecter toute réémergence ou réintroduction. Ce nouveau programme doit s'accompagner de nouveaux outils servant à vérifier l'absence de transmission. L'OMS a donc élaboré un profil de produit cible pour le développement d'un procédé de surveillance transversale de la transmission de *T. b. gambiense* à l'échelle de la population. Ce procédé doit être effectué dans des laboratoires de référence nationaux ou infranationaux et tester simultanément de nombreux échantillons envoyés depuis des régions rurales isolées. Ce profil de produit comporte notamment les caractéristiques suivantes: (i) une procédure simple de collecte d'échantillons; (ii) aucune exigence concernant le respect de la chaîne du froid lors du transfert des échantillons vers les laboratoires de référence; (iii) un niveau élevé de sensibilité et de spécificité; (iv) un haut débit, en grande partie automatisé; (v) de faibles coûts par échantillon lors d'analyses en masse; et enfin, (vi) applicable aux animaux également.

## Resumen

La tripanosomiasis humana africana es una infección parasitaria potencialmente mortal transmitida por la mosca tsetsé en el África Subsahariana. El principal reservorio es el ser humano, y la forma más común está causada por *Trypanosoma brucei gambiense*. El diagnóstico práctico requiere un examen microscópico a cargo de personal con formación específica. Tras más de dos décadas de esfuerzos sostenidos, la incidencia de la enfermedad está disminuyendo considerablemente, y en algunos países históricamente endémicos ya no se detectan casos. La Organización Mundial de la Salud (OMS) se ha fijado como objetivo la eliminación de la transmisión de la tripanosomiasis africana humana gambiense para 2030, es decir, cero casos autóctonos durante al menos cinco años consecutivos. Los países endémicos que alcancen este objetivo deben mantener una vigilancia permanente para detectar la reaparición o reintroducción de la enfermedad. Con esta agenda nueva, se necesitan herramientas nuevas para verificar la ausencia de transmisión. Por consiguiente, la OMS ha elaborado un perfil de producto objetivo en el que se pide el desarrollo de un método para la vigilancia transversal a nivel de población sobre la transmisión de *T. b. gambiense*. El método debe realizarse en laboratorios de referencia nacionales o subnacionales y analizar en paralelo numerosas muestras enviadas desde regiones rurales remotas. Entre otras características, el perfil del producto detalla: (i) un procedimiento sencillo de recogida de muestras; (ii) ningún requisito de cadena de frío para transferir las muestras a los laboratorios de referencia; (iii) alta sensibilidad y especificidad; (iv) alto rendimiento, sustancialmente automatizado; (v) bajo coste por muestra, cuando se analizan en grandes lotes; y (vi) aplicable también en animales.

## ملخص

داء النوم الأفريقي البشري هو عدوى طفيلية مهددة للحياة تنتقل عن طريق ذبابة تسي تسي في جنوب الصحراء الكبرى بأفريقيا. الشكل الأكثر شيوعًا يسببه داء النوم البروسي الغامبي، حيث يكون البشر هم الحامل الرئيسي. يتطلب التشخيص الميداني فحصًا مجهريًا من خلال طرق عسيرة يتم إجراؤها بواسطة فرق عمل مدربة خصيصًا لهذا الغرض. بعد أكثر من عقدين من الجهود المتواصلة، انخفض معدل الإصابة بالمرض بشدة، وبعض الدول التي لها تاريخ سابق في هذا المرض، لم تعد تكتشف أي حالات. استهدفت منظمة الصحة العالمية (WHO) القضاء على انتقال داء النوم الإفريقي البشري الغامبي بحلول عام 2030، والذي تم تعريفه على أنه عدم وجود حالات بين السكان الأصليين لمدة خمس سنوات متتالية على الأقل. يجب على الدول الموبوءة التي تحقق هذا الهدف، أن تحافظ على رصد مخصص لاكتشاف عودة الظهور أو انتقال العدوى. وفي ظل هذا الجدول الجديد للأعمال، هناك حاجة إلى أدوات جديدة للتحقق من غياب انتقال العدوى. لذلك، قامت منظمة الصحة العالمية بتطوير ملف تعريف لمنتج مستهدف يدعو إلى تطوير طريقة للرصد الشامل على مستوى السكان لانتقال داء النوم البروسي الغامبي. تحتاج الطريقة إلى تنفيذها في المعامل المرجعية الوطنية أو دون الوطنية، والاختبار المتوازي للعديد من العينات التي يتم شحنها من المناطق الريفية النائية. من بين الخصائص الأخرى التي يحددها ملف تعريف المنتج: (1) إجراء بسيط لجمع العينات؛ و(2) عدم وجود متطلبات لسلسلة التبريد لنقل العينات إلى المختبرات المرجعية؛ و(3) حساسية وخصوصية عالية؛ و(4) إنتاجية عالية، مؤتمتة إلى حد كبير؛ و(5) تكلفة منخفضة لكل عينة، عند تحليلها على دفعات كبيرة؛ و(6) يمكن تطبيقها أيضًا على الحيوانات.

## 摘要

非洲人类锥虫病是撒哈拉以南非洲地区一种由采采蝇传播的危及生命的寄生虫感染。最常见的感染是由*布氏冈比亚锥虫*引起的，人类是其主要宿主。现场诊断需要由受过专门训练的人员进行繁复的显微镜检查。经过二十多年的持续努力，该疾病的发病率正在大幅下降，并在一些历史上流行该病的国家未再发现新病例。世界卫生组织 (WHO) 的目标是到 2030 年消除非洲人类冈比亚锥虫病的传播，即至少连续五年本土不再出现病例。要实现这一目标，流行该疾病的国家必须针对该病进行专门的监测，以及时发现再次出现或再次发生感染的情况。随着这一新议程的提出，需要新工具来核查是否存在传播的情况。因此，世卫组织制定了一份目标产品简介，呼吁制定一种可以在人群层面对*布氏冈比亚锥虫*病传播进行交叉监测的方法。该方法需要在国家级或次国家级别的参考实验室中进行，并对从偏远农村地区运来的大量样本进行同步测试。产品简介还规定了其他特性：(i) 简单的样本采集程序；(ii) 将样本转移至参考实验室过程中无需冷链保存；(iii) 高灵敏度和特异性；(iv) 高通量、基本自动化处理；(v) 进行大批量分析时每份样本的成本较低；以及 (vi) 也适用于在动物中进行监测。

## Резюме

Африканский трипаносомоз человека представляет собой опасную для жизни паразитарную инфекцию, передаваемую мухой цеце в странах Африки к югу от Сахары. Наиболее распространенная форма вызывается *Trypanosoma brucei gambiense*, основным естественным резервуаром которой является человек. Диагностика в естественных условиях предусматривает проведение микроскопического исследования с использованием трудоемких методов, выполняемых специально обученным персоналом. После более чем двух десятилетий непрерывных усилий показатель заболеваемости сильно снизился, а в некоторых исторически эндемичных странах случаи заболевания больше не выявляются. Всемирная организация здравоохранения (ВОЗ) поставила цель ликвидировать передачу африканского трипаносомоза человека, вызываемого Trypanosoma gambiense, к 2030 году. Под этим подразумевается отсутствие возникновения аутохтонных случаев заболевания в течение как минимум пяти лет подряд. При достижении этой цели в эндемичных странах должен проводиться специальный эпиднадзор для выявления повторной вспышки или реинтродукции заболевания. В соответствии с этой новой повесткой необходимо разработать новые инструменты для проверки отсутствия передачи инфекции. Поэтому ВОЗ был разработан целевой профиль продукта, предусматривающий разработку метода межсекторального эпиднадзора за передачей *T. b. gambiense* на уровне населения. Метод необходимо применять в справочных лабораториях на национальном или субнациональном уровне и параллельно проводить испытания многочисленных образцов, доставленных из отдаленных сельских районов. Среди прочих характеристик в профиле продукта указаны: (i) простая процедура отбора образцов; (ii) отсутствие необходимости передачи образцов в справочные лаборатории; (iii) высокая чувствительность и специфичность; (iv) высокая пропускная способность, значительная автоматизация; (v) низкая стоимость одного образца при анализе больших серий; (vi) применимость также у животных.

## Introduction

Human African trypanosomiasis, also known as sleeping sickness, is a life-threatening parasitic infection transmitted by the tsetse fly. The disease is endemic in sub-Saharan Africa. Having caused devastating epidemics during the 20th century, the incidence of infection has now fallen to historically low levels due to sustained and coordinated efforts over the past 20 years.^1^ The two trypanosome subspecies that cause the disease have distinct epidemiology. *Trypanosoma brucei rhodesiense*, found in eastern and southern Africa, is harboured by wild and domestic animals which constitute its reservoir and is transmitted occasionally to humans. *T. b. gambiense*, found in western and central Africa, has humans as the main reservoir and accounts for about 95% of the total caseload between 2011 and 2020 (32 275 out of 34 096 infections).^1^

The diagnosis of human African trypanosomiasis relies on laboratory techniques because clinical signs and symptoms are unspecific. Field serodiagnostic tests exist only for *T. b. gambiense* and are based on the detection of specific antibodies; thus they are not confirmatory of infection. With the current low disease prevalence, the positive predictive value of serological tests is particularly low.^2^ Field-applicable tools include the card agglutination test for trypanosomiasis, used mainly in active screening by specialized mobile teams, and the rapid diagnostic tests that are more suitable for individual testing at point-of-care. Confirmation of *T. b. gambiense* infection requires microscopic examination of body fluids, necessitating specific training for laboratory staff. The best-performing methods are laborious and reach 85%−95% diagnostic sensitivity when performed by skilled personnel.^3^ Because trypanosomes are identified visually by their characteristic movement, microscopic examination must be done a short time after sampling (less than 1 hour).

Human African trypanosomiasis has been targeted for elimination as a public health problem, defined as a five-year mean of less than 1 case per 10 000 inhabitants in all endemic districts in a given country. This status has been reached in several countries, and has been or will soon be validated by the World Health Organization (WHO).^4^ The next target is the elimination of transmission of gambiense human African trypanosomiasis, defined as zero autochthonous cases for at least five consecutive years.^5^ Countries where the disease is endemic and who reach either of these goals need to maintain dedicated surveillance because of the persisting risk of re-emergence or re-introduction of human African trypanosomiasis.

An unintended consequence of the progress in human African trypanosomiasis elimination is the gradual loss of specialized personnel. This trend is occurring at a time when there is a greater need for large-scale testing of populations at risk to verify the absence of *T. b. gambiense* transmission. The currently available diagnostic tools are complex and resource-intensive to use. Here we describe a target product profile to stimulate the development of high-throughput methods of testing for *T. b. gambiense* that can be performed by non-specialized personnel.

## Methods

### Development process 

The development of this target product profile was led by the WHO Department of Control of Neglected Tropical Diseases following standard WHO guidance for target product profile development. To identify and prioritize diagnostic needs, a WHO Neglected Tropical Diseases Diagnostics Technical Advisory Group was formed, and different subgroups were created to advise on specific neglected tropical diseases, including a subgroup working on the need for innovations in diagnosis of human African trypanosomiasis. This group of independent experts comprised leading international scientists and specialists, including from countries where the disease is endemic. Standard WHO declaration of interest procedures were followed. To identify unmet needs, the subgroup conducted a landscape analysis of the tests that were currently available and those currently being developed. Through meetings and remote consultations, the subgroup developed use cases for hypothetical tools that would fill the main gaps in requirement for testing, and gave the uses an order of priority. The subgroup agreed on a template adapted to the context of human African trypanosomiasis for use in the development of the target product profile. The draft of this target product profile (rated as priority no. 4) underwent several rounds of review by the subgroup members. The Diagnostics Technical Advisory Group members reviewed the resulting version. Draft version 0.1 and a proforma comment form were posted on the WHO website for public consultation over 28 days. WHO then released the current version of the target product profile.^6^

### Use case

The use case was defined as a high-throughput test for verification of elimination of *T. b. gambiense*.

### Technical scope

The technical scope described a method for testing in parallel numerous samples collected in remote rural areas. Ideally, testing should be possible to perform within the country, in national or subnational reference laboratories. At a minimum, testing could be carried out at regional reference laboratories, bearing in mind that shipping samples to other countries is often complex and subject to strict regulations. The test would need to have high sensitivity and specificity. Positive results might need to be characterized further with additional testing, to discard false positives. Ideally, the test would be also applicable in animals, which could help with assessing the circulation of *T. b. gambiense* parasites in a region. The use of the test in vectors would be less important as infection rates in the vector are very low. (In this document, animals refer to non-human vertebrates; vectors refer to tsetse flies.)

### Sampling

Ideally, sampling should be non-invasive. Acceptable sampling methods would be finger-prick or venous blood, serum or plasma (stabilized in whatever carrier), with a stability of 4 weeks at 40 °C, 12 months at 4 °C. Sampling should require a simple specimen collection procedure with no cold-chain requirement to transfer samples to reference laboratories.

After arrival of the specimens in the laboratory, the results should be available in a relatively short time even if thousands of specimens are to be analysed (that is, a high-throughput method). The total cost per specimen, when analysed in batches of hundreds or thousands, should remain low.

To aid interpretation, it should be established for how long an individual can test positive after a *T. b. gambiense* infection has cleared. For example, antibody testing may show a positive result for years after infection,^7^ whereas for molecular tests the clearance of DNA (DNA) and in particular ribonucleic acid (RNA) from blood is within days.^8^ However, persistence of DNA in blood and cerebrospinal fluid has been observed in around one fifth of patients 2 years or more after successful treatment , an effect which remains to be explained.^9^. If specimens from individuals with human African trypanosomiasis history are collected, their data should be documented and interpreted in view of their disease history. Alternatively, former patients could be excluded from sampling.

### Medical need

The incidence of gambiense human African trypanosomiasis has been strongly declining globally, and some countries historically endemic for the disease have not reported new cases for several years, either countrywide or in some previous foci of disease.^1^ Unfortunately, the decline in incidence is often accompanied by a loss of capacity for testing so that case detection becomes increasingly difficult to maintain. There is therefore an increasing need for high-throughput methods that can complement the classic strategies of passive and active screening, each with its own limitations, with appropriate tools for population-level cross-cutting surveillance of *T. b. gambiense* transmission. These tools and methods would allow for testing with more comprehensive coverage of populations considered at risk, and particularly of populations thought to have become risk-free and where absence of transmission needs verification.

## Target product profile

### 1. Intended use

#### Target taxonomy, species, subspecies and type

##### Minimal

*Trypanozoon.*

##### Optimal

*T. b. gambiense.*

##### Notes

Specificity of subspecies is particularly important if vectors or animals are tested.

#### Target population

##### Minimal

Populations (human) at risk of gambiense human African trypanosomiasis.

##### Optimal

Populations (human, animal or vector) at risk of being infected with *T. b. gambiense.*

#### Use of information obtained

##### Minimal

Establish recent circulation of *T. b. gambiense* in humans.

##### Optimal

Establish current circulation of *T. b. gambiense* in humans, animals or vectors.

#### Type of specimen collected

##### Minimal

Minimally invasive specimen.

##### Optimal

Non-invasively collected specimen.

##### Notes

Minimally invasive specimens include finger-prick or venous blood. Non-invasive specimens include saliva, urine or tears. In animals, easy collection avoids the need to capture the animal (faeces) or involves limited discomfort to animal and collector. Invasiveness is not applicable in vectors. Room-temperature storage or shipment is needed.

#### Analyte to be detected

##### Minimal

Antibodies, antigens, whole parasite or nucleic acids.

##### Optimal

Antigens, whole parasite or RNA.

##### Notes

Antibodies may persist in a previously infected and cured patient. RNA is a better marker for current infection than DNA.

#### Nature of the result

##### Minimal

Qualitative.

##### Optimal

Qualitative.

#### Infrastructure level and operating environment

##### Minimal

Laboratory at national level, or even international reference laboratory.

##### Optimal

Laboratory at sub-national or national level.

##### Notes

There may be a trade-off between the difficulties of international shipment of many samples and the set-up of capacity to perform this test in endemic countries.

#### Intended user

##### Minimal

Trained laboratory technician.

##### Optimal

Trained laboratory technician.

### 2. Assay performance characteristics

Assay performance characteristics are relevant to individual patients or population needs.

#### Clinical sensitivity

##### Minimal

> 95%.

##### Optimal

> 99%.

##### Notes

Clinical sensitivity should be at least equal to the most sensitive parasitological tests currently used.

#### Clinical specificity

##### Minimal

> 99%.

##### Optimal

> 99.5%.

In case of a positive result, the test might be combined with confirmatory testing.

#### Analytical specificity or cross reactivity

##### Minimal

*Trypanozoon*-specific for humans, *T. b. gambiense*-specific for animals or vectors. 

##### Optimal

*T. b. gambiense* type 1.

##### Notes

Specificity should be to *T. b. gambiense* type 1 if applied in animals. For human testing *Trypanozoon* might be sufficient to raise concern, yet only infections with *T. b. gambiense type 1* are a threat to gambiense human African trypanosomiasis elimination.

#### Analytical sensitivity

##### Minimal

Corresponding to ≤ 50 parasites/mL.

##### Optimal

Corresponding to ≤ 10 parasites/mL.

##### Notes

Tests detecting antigens or nucleic acid sequences may reach lower detection thresholds than those detecting whole parasites.

#### Repeatability

Repeatability is the intra-reader agreement (different tests, same instruments or environment, same sample, same reader).

##### Minimal

*Κ* > 0.8.

##### Optimal

*Κ* > 0.9.

#### Reproducibility

Reproducibility is the inter-reader agreement (different tests, other instruments or environment, same sample, same reader or different readers).

##### Minimal

*Κ* > 0.8.

##### Optimal

*Κ* > 0.9.

##### Notes

Given the importance of this test in verification of human African trypanosomiasis elimination, repeatability and reproducibility should be as high as possible.

#### Quality control

##### Minimal

Control of functionality, positive and negative controls for batch testing and per run.

##### Optimal

Control of functionality, positive and negative controls for batch testing and per run.

##### Notes

A proficiency panel would be useful.

### 3. Regulatory and normative needs

#### Regulatory approvals and standards

##### Minimal

Test components manufactured according to GMP (ISO 13 485:2016).

##### Optimal

CE marking or other comparable regulatory approval. QMS ISO 13 485:2016.

##### Notes

New, more demanding CE marking rules may entail unrealistic production costs. Alternative registration (e.g. Australian Therapeutic Goods Administration) may be considered. The quality management system should be defined. Dependence on commercial availability.

#### Promotional and marketing material

##### Minimal

Not applicable.

##### Optimal

Not applicable.

### 4. Health-care system needs

### 4.1. Environment description

#### Operating environment

##### Minimal

Can be operated at 10 °C −30 °C at 40%−70% relative humidity.

##### Optimal

Can be operated at 10 °C −40 °C at 10%−88% relative humidity.

##### Notes

The test will be applied in laboratories where temperature and humidity will be well controlled.

#### Workflow requirements

##### Minimal

Specimen preparation in the field in < five steps, minimal need for precision liquid handling, and minimal need for specialized material (generally available or provided in a specimen collection kit). Specimen shipment needs minimal security measures (minimal infection risk) and no or limited cold chain. Testing is fairly well automatized, with < five manual steps; > 100 specimens tested daily.

##### Optimal

Specimen preparation in the field in < two steps, no need for precision liquid handling, and no need for specialized material. Specimen shipment needs no special security measures (no infection risk) nor cold chain. Testing is substantially automatized, with < two manual steps. No need for precision liquid handling; > 500 specimens tested daily.

##### Notes

Analysing pooled samples instead of individual samples could also be considered.

### 4.2. Instrument and device characteristics

#### Instrumentation needed

##### Minimal

Requiring instrumentation and devices that can be implemented at laboratories at national level.

##### Optimal

Requiring instrumentation and devices usually present at laboratories at national or subnational level.

### 4.3. Information and communication technology

#### Test result

##### Minimal

Test results scored visually or by read-out of a device. Test result stable for at least 15 minutes.

##### Optimal

Test results scored by read-out of a device. Test result stable for at least 30 minutes.

#### Recording of results and data capture

##### Minimal

Results are recorded in a computer, either automatically or manually.

##### Optimal

Results recorded in a computer. Integrable into national data and reporting. Test results can be stored for retrospective interpretation (e.g. electronic result, optical density or intensity, electronic image or video). Automatic interpretation of result (positive or negative).

##### Notes

Data should include results and demographics or other information. Data should be exportable to any database if needed. Storage needs may vary per programme.

#### Transmission

##### Minimal

Test results transmitted electronically.

##### Optimal

Data automatically integrated in server databases without need of additional equipment.

##### Notes

Transmission should be adaptable to connectivity. Data format should be compatible with health-care databases such as JavaScript Object Notation (JSON, Ecma International, Geneva, Switzerland) or District Health Information Software 2 (DHIS2, University of Oslo, Oslo, Norway), supporting seamless transmission to them if required.

### 4.4 Reagent and control handling

#### Reagents, storage and packaging

##### Minimal

Reagents stable at 4 °C −8 °C and 40%−88% relative humidity for at least 12 months. Operating instructions and bench aids available. Reagents ready to use, or within 15 minutes, with maximum 5 additional steps.

##### Optimal

Reagents stable at 4 °C −45 °C and 40%−88% relative humidity for ≥ 24 months. One-week transport stress at 50 °C. Transport not needing cold chain. Operating instructions and bench aids available. Reagents ready to use or maximum two additional steps needed.

##### Notes

The stability should consider the time-frame for distribution from manufacturer, passage through customs and local distribution.

### 4.5. Sample handling

#### Sample volumes

##### Minimal

Depending on the type of specimen. For blood (or serum or plasma) ≤ 5 mL.

##### Optimal

Depending on the type of specimen. For blood ≤ 0.07 mL (finger-prick, capillary tube).

##### Notes

Extra specimen material can be collected at the same time for repeat or remote testing if needed. 

#### Specimen collection and processing

##### Minimal

Specific collecting devices provided as a kit. Some specimen processing. Transfer of samples within 1 week. Cold chain recommended but not strict. Thousands of samples can be managed in a reasonable time. Specimen shipment needs minimal security measures (minimal infection risk).

##### Optimal

Routinely used collecting devices, minimal or no specimen processing. Transfer of samples not urgent (e.g. 4 weeks) and not requiring cold chain. Thousands of samples can be managed quickly. Specimen shipment needs no special security measures (no infection risk).

##### Notes

Occasionally, left-over specimens could be preserved and transported under certain conditions.

#### Waste management and biosafety

##### Minimal

Amenable to standard biosafety measures for handling potentially infectious materials. Waste disposal in biosafety bins and sharps containers, following standard guidelines.

##### Optimal

Amenable to standard biosafety measures for handling potentially infectious materials. Waste disposal in biosafety bins and sharps containers, following standard guidelines.

### 4.6. Distribution, training and support

#### Training (sampling)

##### Minimal

Specific training needed (< 4 hours).

##### Optimal

Basic training needed (< 1 hour).

#### Training (laboratory testing)

##### Minimal

Extended specific training needed (7 days).

##### Optimal

Specific training needed (max 1−2 days).

#### Instrument and test supply reliability

##### Minimal

Supply guaranteed for ≥ 5 years after marketing. Manufacturer should replace non-functioning tests or instruments.

##### Optimal

Supply guaranteed for ≥ 7 years after marketing. Manufacturer should replace non-functioning tests or instruments.

#### Service and support response time

##### Minimal

External support available. Support response within 1 week.

##### Optimal

External support available. Support response within 1 day.

### 5. Commercial and sustainability aspects

#### Sustainability 

##### Minimal

Sustainable production.

##### Optimal

Sustainable production.

##### Notes

As it is a non-profitable area, sustainable funding and a production or access innovative model is needed, with donors ensuring affordability. Advocacy is needed.

#### Pricing per sample collected

##### Minimal

≤ 0.5 United States dollars (US$).

##### Optimal

≤ 0.1 US$.

##### Notes

Costs of hardware, shipment of material and human resources are not included here.

#### Pricing per sample tested

##### Minimal

≤ 5 US$.

##### Optimal

≤ 0.5 US$.

##### Notes

All logistics, operational laboratory costs, investments, hardware, shipment of material and salaries, are not included here. Molecular methods cost is a trade-off with clinical sensitivity.

## Conclusion

This target product profile was developed by a WHO advisory group of independent experts working on gambiense human African trypanosomiasis, comprising leading international scientists and specialists, including from endemic countries. The product profile is intended to promote the development of a new test that would be most useful in the agenda of human African trypanosomiasis elimination, including in the post-elimination phase. Among other characteristics, the product profile specifies: (i) a simple specimen collection procedure; (ii) no cold-chain requirement for transfer of specimens to reference laboratories; (iii) high sensitivity and specificity; (iv) high-throughput, substantially automatized; (v) low cost per specimen, when analysed in large batches; and (vi) applicable also in animals.
